# 543 Identifying Barriers and Enablers to Translating Scientific Evidence into Burns First Aid Practices

**DOI:** 10.1093/jbcr/irae036.177

**Published:** 2024-04-17

**Authors:** Yvonne M Singer, Maleea Holbert, Kevin Mackey, John S Rose, Tina L Palmieri, Bronwyn R Griffin

**Affiliations:** Griffith University, Melbourne, Victoria; Queensland Children's Hospital, South Brisbane, Queensland; Sacramento Fire Department, Sacramento, CA; UC Davis Medical Centre, Sacramento, CA; UC Davis, Shriners Children's Northern California, Sacramento, CA; Griffith University, Brisbane, Queensland; Griffith University, Melbourne, Victoria; Queensland Children's Hospital, South Brisbane, Queensland; Sacramento Fire Department, Sacramento, CA; UC Davis Medical Centre, Sacramento, CA; UC Davis, Shriners Children's Northern California, Sacramento, CA; Griffith University, Brisbane, Queensland; Griffith University, Melbourne, Victoria; Queensland Children's Hospital, South Brisbane, Queensland; Sacramento Fire Department, Sacramento, CA; UC Davis Medical Centre, Sacramento, CA; UC Davis, Shriners Children's Northern California, Sacramento, CA; Griffith University, Brisbane, Queensland; Griffith University, Melbourne, Victoria; Queensland Children's Hospital, South Brisbane, Queensland; Sacramento Fire Department, Sacramento, CA; UC Davis Medical Centre, Sacramento, CA; UC Davis, Shriners Children's Northern California, Sacramento, CA; Griffith University, Brisbane, Queensland; Griffith University, Melbourne, Victoria; Queensland Children's Hospital, South Brisbane, Queensland; Sacramento Fire Department, Sacramento, CA; UC Davis Medical Centre, Sacramento, CA; UC Davis, Shriners Children's Northern California, Sacramento, CA; Griffith University, Brisbane, Queensland; Griffith University, Melbourne, Victoria; Queensland Children's Hospital, South Brisbane, Queensland; Sacramento Fire Department, Sacramento, CA; UC Davis Medical Centre, Sacramento, CA; UC Davis, Shriners Children's Northern California, Sacramento, CA; Griffith University, Brisbane, Queensland

## Abstract

**Introduction:**

Evidence shows that 20 minutes of cool running water applied within 3 hours of burn injury (20CRW) improves patient outcomes including reduced odds of requiring skin grafting or hospitalisation. Study investigators are undertaking an implementation research to translate 20CRW into clinical practices of County Fire and Emergency Medical Services (EMS) and the Burn Center’s Emergency Department (ED). As part of this research, we undertook a sub-project, with local EMS and ED clinical champions. to identify barriers and enablers to 20CRW practice change and co-design implementation strategies to address them.

**Methods:**

Online surveys were developed and disseminated to EMS and ED clinicians and focus groups involving EMS and ED clinician champions were conducted. Results were deductively coded using the Consolidated Framework for Implementation Research (CFIR) and then mapped to the Expert Recommendations for Implementing Change (ERIC) matching tool to identify potential implementation strategies. Findings from the surveys, focus groups and CFIR-ERIC mapped strategies were then fedback to clinical champions to achieve consensus and inform the subsequent co-design of tailored implementation strategies to address them.

**Results:**

A total of 204 EMS and 96 ED clinicians completed the survey, and 10 EMS and 9 ED clinical champions participated in four focus group sessions. Multiple interrelated barriers and enablers related to clinicians, patients, the 20CRW intervention, organisations and environment were identified that covered four of the CFIR domains. Most clinicians were unaware of 20CRW (>80%). Whilst 82% of ED and 56% of EMS clinicians considered 20CRW an acceptable practice change, 60% and 65% of ED and EMS clinicians respectively, deemed it different to current practices. The relative advantage of delivering 20CRW to patients with large burns with risks of hypothermia and/or other urgent care needs was raised; access to water was a logistical challenge in the variable pre-hospital environment and the structural design of the ED and ED cubicles; contaminated water in the prehospital environment and the risk of wound contamination were also raised.

Development of multiple tailored implementation strategies are underway including guidelines with patient exclusion criteria and advice regarding water sources, recruitment and training of implementation champions, educational resources and fact sheets, and purchasing of equipment for cooling enroute to hospital and in ED cubicles.

**Conclusions:**

Translating evidence into sustainable clinical practice change is complex and requires more than new guidelines, knowledge and skills. Validated implementation science methodologies can assist in identifying barriers and enablers to sustainable practice changes.

**Applicability of Research to Practice:**

Findings of this research will inform the translation of 20CRW evidence into County EMS and ED guidelines and clinical practices.

